# Pharmacokinetics of orally administered single‐dose ponazuril in cats

**DOI:** 10.1111/jvp.13047

**Published:** 2022-03-20

**Authors:** Catherine Burlison, Sherry Cox, Joseph Smith, Jennifer Stokes, Jacqueline C. Whittemore, Becky DeBolt

**Affiliations:** ^1^ Department of Small Animal Clinical Sciences College of Veterinary Medicine The University of Tennessee Knoxville Tennessee USA; ^2^ Department of Biomedical and Diagnostic Sciences College of Veterinary Medicine The University of Tennessee Knoxville Tennessee USA; ^3^ Department of Large Animal Clinical Sciences College of Veterinary Medicine The University of Tennessee Knoxville Tennessee USA; ^4^ Animal Emergency and Specialty Center of Knoxville Knoxville Tennessee USA

**Keywords:** cats, coccida, coccidiosis, ponazuril, shelter

## Abstract

Cats and kittens in animal shelters and catteries regularly suffer from severe gastrointestinal coccidiosis, which can be fatal, and there are no drugs labeled for feline coccidiosis in the United States. Ponazuril, a triazine‐class drug, is increasingly used at a dose of 50 mg/kg/d, orally, for three to five days in shelter environments for coccidiosis. A single oral dose of ponazuril paste 15% (Marquis^®^; Merial) at 50 mg/kg was administered to six healthy adult cats. Sample analysis was completed via high‐performance liquid chromatography. Plasma concentrations peaked at 7.49 ± 2.06 µg/ml at 14.67 ± 7.45 hr post‐administration. This study shows that ponazuril achieved a plasma concentration that inhibits growth of similar organisms after a single oral dose in cats. Further studies are necessary to optimize dosing for the treatment of clinical coccidiosis in cats.

## 1 INTRODUCTION

Intestinal coccidiosis is a common cause of morbidity and mortality in kittens in animal shelters, and its pathogenesis remains poorly understood. (Dubey et al., 2009) Coccidia are obligate intracellular parasites routinely found in the intestinal tracts of mammals. Like many infectious diseases, age and living conditions appear to be the most influential factors in the prevalence of *Cystoisospora felis and Cystoisospora rivolta*, the most common species of coccidia in cats. Neonates and kittens less than three months old are the most susceptible to experimental infections and have the highest rate of natural infection. (Barutzki & Schaper, 2011; Dubey et al., 2009; Shah, 1971) Breeding facilities and shelters, where the cat populations are dense and the environments could be unhygienic, have higher prevalence of coccidia infected animals. (Dubey et al., 2009; Lloyd & Smith, 2001) Studies in shelters in North America have described prevalence rates from 14% to 21%. (Hoggard et al., 2018, 2019; Lucio‐Forster & Bowman, 2011; Villeneuve et al., 2015; Wyrosdick et al., 2017) Sources of stress such as weaning, shipping, and change of ownership also contribute to development of symptomatic disease. While many cats do not exhibit clinical signs of coccidiosis, some develop mucoid or hemorrhagic diarrhea leading to weight loss and dehydration. (Dubey et al., 2009; Lloyd & Smith, 2001) Vomiting, anorexia, lethargy, hypoproteinemia, and death or euthanasia occur with severe infections. (O’Brien et al., 2002).

Intestinal coccidiosis is difficult for shelters to manage as individuals most susceptible to clinical disease also are vulnerable to other life‐threatening illnesses. The only Food and Drug Administration (FDA)‐approved drug for treatment of coccidiosis in a small animal is the coccidiostatic drug sulfadimethoxine, it is administered for five to 21 days for coccidia, which makes it intensive from a time and economic standpoint. Lloyd and O’Brien reported in the early 2000s that toltrazuril reduces coccidia oocyst shedding and lessens diarrhea in kittens. (Lloyd & Smith, 2001; O’Brien et al., 2002) Dosing recommendations have ranged from only one dose, one dose followed by a second dose five days later, to daily doses for three or five days. Unfortunately, toltrazuril is not available in the United States of America (US). Ponazuril, a metabolite of toltrazuril, is available in the United States, which is commonly used even though it can be harder to accurately dose due to the concentration of the commercially available product. A recent study by Litster demonstrated the highest reduction of fecal oocysts when naturally infected shelter cats were dosed with 50 mg/kg of ponazuril for three consecutive days. This is a much higher dosage than recommended for other species. Of the cats, 12.5% had detectable oocysts at days three and four post‐treatment. (Litster et al., 2014) It is unknown whether persistent shedding was due to inadequate absorption and, thus, subtherapeutic blood concentrations in some cats. If so, a higher dosage might be more efficacious in treating gastrointestinal coccidiosis. The objective of this study was to obtain pharmacokinetic parameters for a single oral administration of ponazuril (50 mg/kg) in healthy adult cats.

## 2 MATERIALS AND METHODS

### 2.1 Experimental subjects

Six healthy, female adult, purpose‐bred domestic short hair cats from a research colony at the University of Tennessee, Knoxville, were used. Cats were excluded if abnormalities were present on laboratory diagnostics performed two months prior to the study (CBC, serum biochemistry profile, and urinalysis), or if there were any physical examination findings suggestive of systemic or gastrointestinal disease including poor hair coat, low body or muscle condition score (<5/9 or <3/3, respectively), or abnormalities identified on abdominal palpation or thoracic auscultation. The cats weighed 3.58–5.08 kg with a mean of 4.53kg. The study was approved by the Institutional Animal Care and Use Committee at the University of Tennessee, Knoxville (Protocol #2783‐0920).

Studies using six animals allow the acquisition of precise preliminary estimates of the average pharmacokinetic parameters. (Riviere, 2011).

Twenty‐four hours prior to ponazuril administration, 24G long line sampling catheters (MILA International) were placed after intramuscular sedation with 40 μg/kg dexmedetomidine (Dexdomitor^®^; Orion Co.) and 5 mg/kg ketamine (Zetamine^®^; Vet One). The following day (24 hr later), the cats were administered 50 mg/kg of ponazuril paste 15% (Marquis^®^; Merial), orally and monitored for complete ingestion. Ponazuril was weighed to the nearest microgram to ensure dose accuracy. Sampling catheters were removed within two days of placement due to decreased blood sampling frequency or lack of patency. Blood was collected at time 0, 15, and 30 minutes, then 1, 2, 4, 8, 12, 24, 48, 72, 96, 120, 144, 168, 192, 216, 240, 264, 288, 312, and 336 hr. These time points were chosen based on clearance times observed in other species. (Dirikolu et al., 2009; Prado et al., 2011; Zou et al., 2014) Most samples consisted of 1ml of blood; no more than 24 ml of blood was taken from one cat over the 2 week period. For samples taken via catheter, a 3 ml sample was withdrawn prior to collection of the sample for testing, which was returned prior to flushing the catheter to limit the total volume of blood removed from each cat. The location of venipuncture for samples taken after catheters were removed alternated on the right and left sides from lateral saphenous, medial saphenous, cephalic, and jugular veins. Plasma was spun at 3,000 *g* for 15 min and frozen at −80ºC and assayed within 2 weeks of collection. Cats were monitored for lethargy, restlessness, change in appetite, vomiting, and diarrhea twice daily throughout the study.

### 2.2 Extraction method

Ponazuril was extracted from plasma samples using a liquid‐liquid extraction method. (Cox et al., 2021) Previously frozen plasma samples were thawed, vortex‐mixed, and 100 µl of plasma was transferred to a 13 × 100 mm screw top tube followed by 10 µl of diclazuril (internal standard, 100 µg/ml) and 2 ml chloroform. The tubes were rocked for 15 min and then centrifuged for 20 min at 1000 *g*. The organic layer was transferred to a clean tube and evaporated to dryness with nitrogen gas. Samples were reconstituted in 250 µl of mobile phase and 100 µl was analyzed.

### 2.3 Chromatographic conditions and apparatus

The system consisted of a 2695 separations module, a 2487 UV absorbance detector, and a computer equipped with Empower software (Waters). The compounds were separated on a Symmetry RP_18_ (4.6 × 150 mm, 5 µm) column with a Symmetry Shield RP_18_ (3.8 mm × 5 mm × 5µm) guard column. The mobile phase was a mixture of 0.1% formic acid in water and acetonitrile (50:50 v/v). Absorbance was measured at 254 nm with a flow rate of 1.1 ml/min and the column temperature was maintained at 23°C.

### 2.4 Calibration

Calibration plasma samples were obtained from six different cats not treated with ponazuril, to verify the possibility of any interfering matrix compounds near the retention time of ponazuril, and were prepared exactly as study plasma samples. The standard curve was composed of 0.1, 0.25, 0.5, 1, 2.5, 5, 10 and 25 μg/ml concentrations, which were chosen based on expected results of study samples. Standard curves for plasma analysis were prepared by fortifying untreated, pooled plasma with ponazuril to produce a linear concentration range of 0.1 – 25 µm/ml.

### 2.5 Pharmacokinetic analysis

Computer software (Phoenix 64 WinNonlin 8.1, Pharsight Corp) was used to calculate pharmacokinetic parameters for ponazuril, including elimination half‐life (t½), elimination rate constant (λz), maximum plasma concentration (C_max_), time to maximum plasma concentration (t _max_), area under the plasma concentration‐time curve from time 0 to last point (AUC_0‐last_), area under the plasma concentration‐time curve from time 0 to infinity (AUC_0‐∞_), percent of the AUC_0‐∞_ extrapolated to infinity (AUC_extrap_), and mean residence time (MRT_0‐∞_) using non‐compartmental analysis. Variability in pharmacokinetic parameters was expressed as the standard deviation. In the case of the half‐life, the harmonic mean and pseudostandard deviation were used instead.

## 3 RESULTS

The average recovery of ponazuril for the quality control (QC) samples (0.3, 7.5, and 15 µg/ml) and the LLOQ was 101% ± 0.5%. This was obtained for the liquid‐liquid extraction method from feline plasma using a reversed phase high performance liquid chromatography method. The intra‐assay variability for the QC samples ranged from 3.7% to 9% while the accuracy was 100%. The inter‐assay variability ranged from 4.9% to 7.6% and the accuracy ranged from 96% to 104%. The lower limit of quantification (LLOQ) was 0.1 µg/ml. The accuracy and precision for the method was considered acceptable based on the FDA 2018 guidelines. (Food and Drug Administration, 2018, 2019)

**FIGURE 1:**
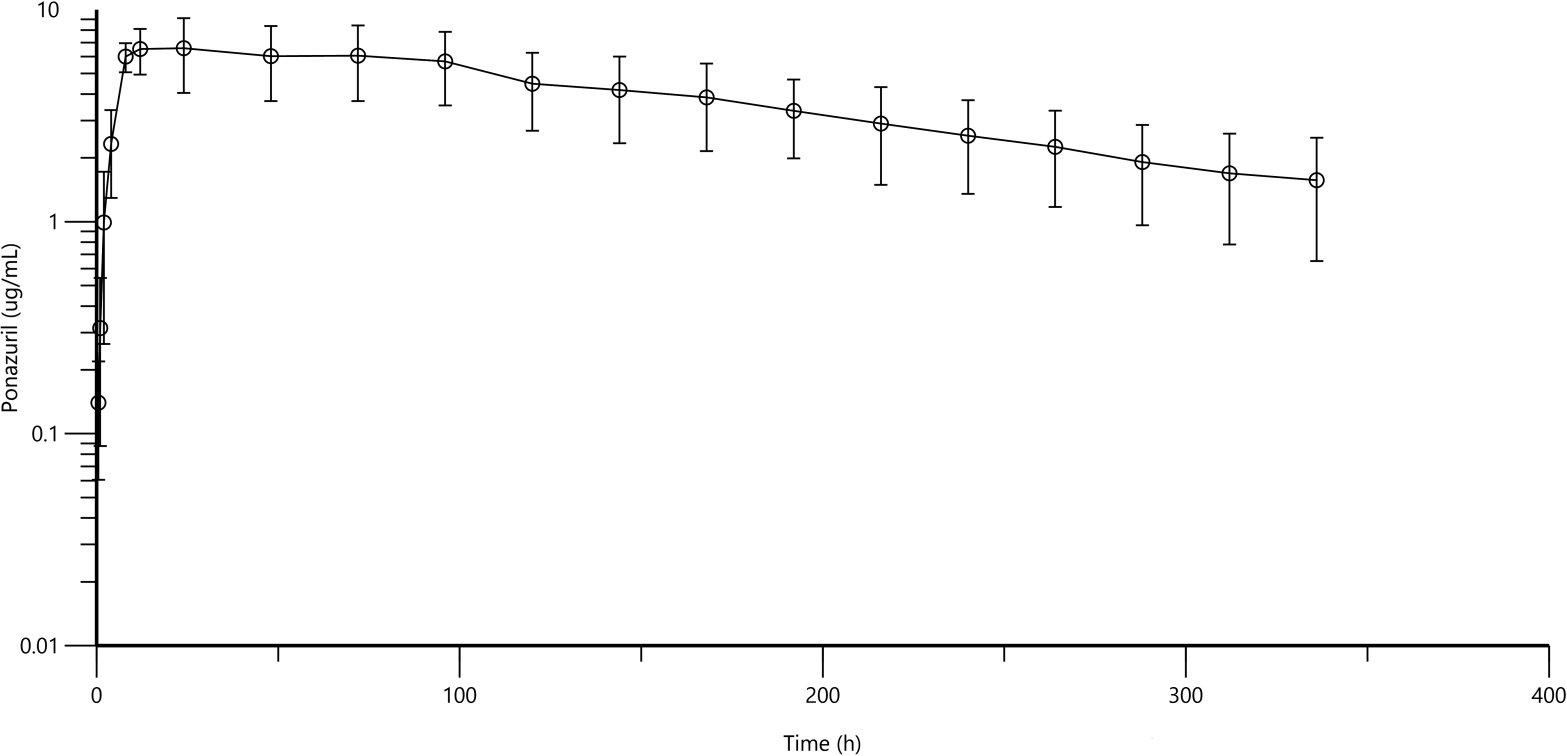
Mean plasma concentration‐time curve for ponazuril following oral administration of a single dose (50 mg/kg) to 6 healthy cats. Values reported are mean ± SD

Mean plasma pharmacokinetic parameters and the plasma concentration‐time profile are shown in Table 1 and Figure 1. Analysis of plasma samples collected following oral administration of a single dose of ponazuril (50 mg/kg) to six healthy cats confirmed systemic absorption of the drug. Plasma samples obtained at time 0 minutes had no detectable concentrations of ponazuril. Peak plasma concentration of 7.49 ± 2.06 µg/ml occurred at 14.67 ± 7.45 hr. Plasma concentrations declined to 1.57 ± 0.92 µg/ml with an average elimination half‐life of ~136 hr. Plasma concentrations were still detectable at 336 hr after ponazuril administration. No side effects were observed.

**TABLE 1:** Pharmacokinetic parameters (mean ± SD) in cats following oral administration of 50 mg/kg ponazuril (*n* = 6)

Pharmacokinetic parameter	Ponazuril mean ± SD
Elimination half‐life t½ (h)*	136 ± 48
Elimination rate constant λz (1/h)	0.006 ± 0.002
t_max_ (h)	14.67 ± 7.45
C_max_ (µg/ml)	7.49 ± 2.06
AUC_0–last_ (h∙µg/ml)	1302 ± 490
AUC_0–∞_ (h∙µg/ml)	1649 ± 733
AUC_extrap_ (%)	18.6 ± 9.9
MRT_0–∞_(h)	208 ± 67

Note

Elimination half‐life (t½), elimination rate constant (λz), maximum plasma concentration (C_max_), time to maximum plasma concentration (t_max_)_,_ area under the plasma concentration‐time curve from time 0 to last point (AUC_0‐last_), area under the plasma concentration‐time curve from time 0 to infinity (AUC_0‐∞_), percent of the AUC_0‐∞_ extrapolated to infinity (AUC_extrap_), mean residence time (MRT_0‐∞_).

## 4 DISCUSSION

Ponazuril, (1‐methyl‐3‐[3‐methyl‐4‐(4‐trifluoromethanesulfonyl‐phenoxy)‐phenyl]‐[1,3,5]‐triaziane‐2,4,6‐trione), is a triazine derivative coccidiocidal drug that has been studied in numerous species including swine, llamas, cattle, goats, horses, and dogs. (Charles et al., 2007; Dirikolu et al., 2009; Furr & Kennedy, 2000; Love et al., 2015; Prado et al., 2011; Zou et al., 2014) It is available in the United States as Marquis^®^ Paste, and is labeled to treat *Sarcocystis neurona* infection in horses. Ponazuril is an effective anti‐coccidial medication in chickens, calves, and buffalo. (Epe et al., 2005; Ghanem et al., 2008; Laczay et al., 1995) Previous pharmacokinetic studies have determined absorption of single‐dose oral administration of ponazuril in cattle, goats, horses, llamas, and swine. (Dirikolu et al., 2009; Furr & Kennedy, 2000; Love et al., 2015; Prado et al., 2011; Zou et al., 2014) Pharmacokinetic parameters vary dramatically by species. Both Dirikolu, et al. and Prado, et al. proposed that the variability of half‐life among species may reflect a difference in the rate of oral absorption rather than elimination rate. (Dirikolu et al., 2009; Prado et al., 2011) The rate of absorption can be affected by binding to food in the gastrointestinal tract. The earliest detection of plasma ponazuril above the LLOQ occurred at 30 min in 50% of the subjects, with detection in all cats at 1 hr. The t _max_ was 14.67 ± 7.45 hr, indicating absorption rate was much faster in cats than llamas, goats, swine, and cattle; (Dirikolu et al., 2009; Love et al., 2015; Prado et al., 2011; Zou et al., 2014) however, the determination of t_max_ can be dependent on sampling schedule and the clinical significance of this difference is unknown. The C_max_ ranged from 5.4 to 10.4 µg/ml with a mean of 7.49 ± 2.06 µg/ml, which is higher than cattle or swine but lower than llamas, horses, and goats. (Dirikolu et al., 2009; Furr & Kennedy, 2000; Love et al., 2015; Prado et al., 2011; Zou et al., 2014) It should be noted for the C_max_ comparisons that these other studies used lower dosages than the cats in this study, with llamas receiving 20 mg/kg, goats and horses receiving 10 mg/kg, and cattle and pigs receiving 5 mg/kg. The mean t½ of ponazuril in cats was similar to that of goats, llamas, and swine, whereas it was greater than two‐fold longer than in cattle or horses. (Dirikolu et al., 2009; Furr & Kennedy, 2000; Love et al., 2015; Prado et al., 2011; Zou et al., 2014).

Limitations of this study include the inability to study bioavailability and the sampling timeframe. An injectable formulation of ponazuril is not available; therefore, the oral bioavailability of ponazuril in cats cannot be determined from our study; however, future studies could consider preparing ponazuril in a suitable vehicle for intravenous administration. Sample collection was stopped 14 days after ponazuril administration, at which point ponazuril was still measurable. When the last measured concentrations are above the assay lower limit of quantification, parameters such as half‐life and mean residence time can be artifactually elevated, (Smith et al., 2018) as the ability to detect lower concentrations for a longer period of time will extend elimination half‐life and mean residence time in a mathematically correct manner, but not in a clinically relevant one. Future studies investigating the pharmacokinetics of ponazuril in cats should employ a longer sampling regimen to more completely capture elimination kinetics. Although we did estimate the apparent elimination half‐life, a large portion of the AUC was extrapolated. This suggests that samples should have been collected for an extended timeframe to more accurately estimate the elimination half‐life.

With no in vitro studies exploring the sensitivity of *C*. *felis* or *C*. *rivolta* to ponazuril, it is not possible to determine if the anecdotal dosage needed to decrease oocyst shedding in cats (50 mg/kg) is due to properties of feline metabolism of the drug or decreased sensitivity of *Cystoisospora spp* to the drug itself. With this dosage, a plasma concentration was reached that has been shown to inhibit growth in similar organisms, in vitro, and is effective against Apicomplexans and used therapeutically in other species. (Alnassan et al., 2013; Furr et al., 2001; Gibbons et al., 2016; Joachim & Mundt, 2011; Jonsson et al., 2011; Kritzner et al., 2002; Mitchell et al., 2005; Redrobe et al., 2010) Robust pharmacodynamic studies, in which repeated dosing pharmacokinetics are paired with assessment of efficacy in the shelter environment, must be completed to validate the clinical dosage and efficacy of ponazuril against intestinal coccidiosis in cats.

## CONFLICT OF INTEREST

The authors disclose no conflict of interest.

## AUTHOR CONTRIBUTION

CDB contributed to sample collection, data analysis, and manuscript writing. SC led analytical method development, sample and data analysis, and contributed to study design and manuscript preparation. JSS was involved in sample collection, data analysis, and manuscript preparation. JES contributed to overall study execution and manuscript preparation. JCW contributed to overall study execution and manuscript preparation. BKD was involved in study design, sample collection, data analysis, and manuscript preparation. All authors have read and approved the final manuscript.

## ETHICAL APPROVAL

The authors confirm that the ethical policies of the journal, as noted on the journal's author guidelines page, have been adhered to and the appropriate ethical review committee approval was received. The authors confirm that they have adhered to US standards for the protection of animals used for scientific purposes. This study was approved by the Institutional Animal Care and Use Committee at the University of Tennessee, Knoxville (Protocol #2783‐0920).

## Data Availability

The data that support the findings of this study are available from the corresponding author upon reasonable request.

## References

[jvp13047-bib-0001] Alnassan, A. A. , Shehata, A. A. , Kotsch, M. , Schrödl, W. , Krüger, M. , Daugschies, A. , & Bangoura, B. (2013). Efficacy of early treatment with toltrazuril in prevention of coccidiosis and necrotic enteritis in chickens. Avian Pathology, 42(5), 482–490. 10.1080/03079457.2013.823476 23941631

[jvp13047-bib-0002] Barutzki, D. , & Schaper, R. (2011). Results of parasitological examinations of faecal samples from cats and dogs in Germany between 2003 and 2010. Parasitology Research, 109(S1), 45–60. 10.1007/s00436-011-2402-8 21739375

[jvp13047-bib-0004] Charles, S. D. , Chopade, H. M. , Ciszewski, D. K. , Arther, R. G. , Settje, T. L. , & Reinemeyer, C. R. (2007). Safety of 5% ponazuril (toltrazuril sulfone) oral suspension and efficacy against naturally acquired *Cytoisospora ohioensis‐*like infection in beagle puppies. Parasitology Research, 2017(101), S137–S144.

[jvp13047-bib-0005] Cox, S. , Harvill, L. , Singleton, S. , Bergman, J. , & DeBolt, B. (2021). Bioanalytical RP‐HPLC method for the determination of ponazuril in plasma. Biomedical Chromatography. 35(12), e5210. 10.1002/bmc.5210. Epub ahead of print. 34216023

[jvp13047-bib-0007] Dirikolu, L. , Yohn, R. , Garrett, E. F. , Chakkath, T. , & Ferguson, D. C. (2009). Detection, quantifications and pharmacokinetics of toltrazuril sulfone (Ponazuril^®^) in cattle. Journal of Veterinary Pharmacology and Therapeutics, 32(3), 280–288. 19646093 10.1111/j.1365-2885.2008.01039.x

[jvp13047-bib-0008] Dubey, J. P. , Lindsay, D. S. , & Lappin, M. R. (2009). Toxoplasmosis and other intestinal coccidial infections in cats and dogs. Veterinary Clinics of North America: Small Animal Practice, 39(6), 1009–1034. 10.1016/j.cvsm.2009.08.001 19932360

[jvp13047-bib-0009] Epe, C. , von Samson‐Himmelstjerna, G. , Wirtherle, N. , von der Heyden, V. , Welz, C. , Beening, J. , Radeloff, I. , Hellmann, K. , Schneider, T. , & Krieger, K. (2005). Efficacy of toltrazuril as a metaphylactic and therapeutic treatment of coccidiosis in first‐year grazing calves. Parasitology Research, 97(S1), S127–S133. 16228269 10.1007/s00436-005-1456-x PMC7087692

[jvp13047-bib-0010] Furr, M. , & Kennedy, T. (2000). Cerebrospinal fluid and blood concentrations of toltrazuril 5% suspension in the horse after oral dosing. Veterinary Theriogenology, 1(2), 125–132. 19757559

[jvp13047-bib-0011] Furr, M. , Kennedy, T. , MacKay, R. , Reed, S. , Andrews, F. , Bernard, B. , Bain, F. , & Byars, D. (2001). Efficacy of ponazuril 15% oral paste as a treatment for equine protozoal myeloencephalitis. Veterinary Therapeutics: Research in Applied Veterinary Medicine, 2, 215–222. 19746664

[jvp13047-bib-0013] Ghanem, M. M. , Radwaan, M. E. , Moustafa, A. M. , & Ebeid, M. H. (2008). Comparative therapeutic effect of toltrazuril, sulphadimidine and amprolium on *Eimeria bovis* and *Eimeria zuernii* given at different times following infection in buffalo calves (*Bubalus bubalis*). Preventive Veterinary Medicine, 84(1–2), 161–170. 18262668 10.1016/j.prevetmed.2007.12.013

[jvp13047-bib-0014] Gibbons, P. , Love, D. , Craig, T. , & Budke, C. (2016). Efficacy of treatment of elevated coccidial oocyst counts in goats using amprolium versus ponazuril. Veterinary Parasitology, 218, 1–4. 10.1016/j.vetpar.2015.12.020 26872920

[jvp13047-bib-0015] Food and Drug Administration (2018). Guidance for industry bioanalytical method validation. Biopharmaceutics. U.S. Department of Health and Human Services, Food and Drug Administration, Center for Drug Evaluation and Research (CDER), Center for Veterinary Medicine (CVM). https://www.fda.gov/media/70858/download

[jvp13047-bib-0016] Hoggard, K. R. , Jarriel, D. M. , Bevelock, T. J. , & Verocai, G. G. (2019). Prevalence survey of gastrointestinal and respiratory parasites of shelter cats in northeastern Georgia, USA. Veterinary Parasitology Regional Studies Reports, 16, 100270. 31027603 10.1016/j.vprsr.2019.100270

[jvp13047-bib-0017] Joachim, A. , & Mundt, H. C. (2011). Efficacy of sulfonamides and Baycox(^®^) against Isospora suis in experimental infections of suckling piglets. Parasitology Research, 109(6), 1653–1659. 10.1007/s00436-011-2438-9 21556685

[jvp13047-bib-0018] Jonsson, N. N. , Piper, E. K. , Gray, C. P. , Deniz, A. , & Constantinoiu, C. C. (2011). Efficacy of toltrazuril 5 % suspension against Eimeria bovis and Eimeria zuernii in calves and observations on the associated immunopathology. Parasitology Research, 109(Suppl 1), S113–S128. 10.1007/s00436-011-2408-2 21739381

[jvp13047-bib-0019] Kritzner, S. , Sager, H. , Blum, J. , Krebber, R. , Greif, G. , & Gottstein, B. (2002). An explorative study to assess the efficacy of toltrazuril‐sulfone (ponazuril) in calves experimentally infected with Neospora caninum. Annals of Clinical Microbiology and Antimicrobials, 1, 4. 12437777 10.1186/1476-0711-1-4 PMC149379

[jvp13047-bib-0020] Laczay, P. , Vörös, G. , & Semjén, G. (1995). Comparative studies on efficacy of sulphachlorpyrazine and toltrazuril for the treatment of caecal coccidiosis in chickens. International Journal of Parasitology, 25(6), 753–756. 7657461 10.1016/0020-7519(94)00180-v

[jvp13047-bib-0021] Litster, A. L. , Nichols, J. , Hall, K. , Camp, J. , & Mohamed, A. S. (2014). Use of ponazuril paste to treat coccidiosis in shelter‐housed cats and dogs. Veterinary Parasitology, 202(3–4), 319–325. 10.1016/j.vetpar.2014.03.003 24679485

[jvp13047-bib-0022] Lloyd, S. , & Smith, J. (2001). Activity of toltrazuril and diclazuril against *Isospora* species in kittens and puppies. The Veterinary Record, 148, 509–511. 11345994 10.1136/vr.148.16.509

[jvp13047-bib-0023] Love, D. , Gibbons, P. , Fajt, V. , & Jones, M. (2015). Pharmacokinetics of single‐dose oral ponazuril in weanling goats. Journal of Veterinary Pharmacology and Therapeutics, 39(3), 305–308. 26542450 10.1111/jvp.12273

[jvp13047-bib-0024] Lucio‐Forster, A. , & Bowman, D. D. (2011). Prevalence of fecal‐borne parasites detected by centrifugal flotation in feline samples from two shelters in upstate New York. Journal of Feline Medicine and Surgery, 13(4), 300–303. 21334238 10.1016/j.jfms.2010.12.013 PMC10832825

[jvp13047-bib-0025] Mitchell, S. M. , Zajac, A. M. , Davis, W. L. , Kennedy, T. J. , & Lindsay, D. S. (2005). The effects of ponazuril on development of apicomplexans in vitro. The Journal of Eukaryotic Microbiology, 52(3), 231–235. 15926999 10.1111/j.1550-7408.2005.00029.x

[jvp13047-bib-0027] O’Brien, C. R. , Pope, S. E. , & Malik, R. (2002). Vomiting, diarrhoea and inappetence in a young cat with hypoproteinaemia. Australian Veterinary Journal, 80(9), 544–546. 10.1111/j.1751-0813.2002.tb11032.x 12398315

[jvp13047-bib-0028] Prado, M. E. , Ryman, J. T. , Boileau, M. J. , Martin‐Jimenez, T. , & Meiborm, B. (2011). Pharmacokinetics of ponazuril after oral administration to healthy llamas (Lama glama). American Journal of Veterinary Research, 72(10), 1386–1389. 21962282 10.2460/ajvr.72.10.1386

[jvp13047-bib-0029] Redrobe, S. P. , Gakos, G. , Elliot, S. C. , Saunders, R. , Martin, S. , & Morgan, E. R. (2010). Comparison of toltrazuril and sulphadimethoxine in the treatment of intestinal coccidiosis in pet rabbits. Veterinary Record, 167(8), 287–290. 10.1136/vr.c3453 20729515

[jvp13047-bib-0030] Riviere, J. E. (2011). Comparative pharmacokinetics: Principles, techniques and applications (pp. 295–313). Wiley‐Blackwell.

[jvp13047-bib-0031] Shah, H. L. (1971). The life cycles of *Isospora felis* Wenyon, 1923, a coccidium of the cat. The Journal of Protozoology, 18, 3–17. 5547073 10.1111/j.1550-7408.1971.tb03271.x

[jvp13047-bib-0032] Smith, J. S. , Coetzee, J. F. , Fisher, I. W. G. , Borts, D. J. , & Mochel, J. P. (2018). Pharmacokinetics of fentanyl citrate and norfentanyl in Holstein calves and effect of analytical performances on fentanyl parameter estimation. The Journal of Pharmacology and Therapeutics, 41(4), 555–561. 10.1111/jvp.12501 29603262

[jvp13047-bib-0033] Villeneuve, A. , Polley, L. , Jenkins, E. , Schurer, J. , Gilleard, J. , Kutz, S. , Conboy, G. , Benoit, D. , Seewalk, W. , & Gagné, F. (2015). Parasite prevalence in fecal samples from shelter dogs and cats across the Canadian provinces. Parasites and Vectors, 8(1), 281. 26013283 10.1186/s13071-015-0870-x PMC4451884

[jvp13047-bib-0034] Wyrosdick, H. M. , Chapman, A. , Martinez, J. , & Schaefer, J. J. (2017). Parasite prevalence survey in shelter cats in Citrus County, Florida. Veterinary Parasitology: Regional Studies Reports, 10, 20–24. 31014592 10.1016/j.vprsr.2017.07.002

[jvp13047-bib-0035] Zou, M. , Guo, G. , Zhao, Y. , & Zhang, Q. (2014). Detection, quantifications, and pharmacokinetics of ponazuril in healthy swine. The Journal of Pharmacology and Therapeutics, 37(6), 598–602. 10.1111/jvp.12126 24731142

